# Associations Between Endoscopic Primary Prophylaxis and Rebleeding in Liver Cirrhosis Patients with Esophagogastric Variceal Bleeding

**DOI:** 10.3389/fsurg.2022.925915

**Published:** 2022-07-12

**Authors:** Yanying Gao, Haixia Yuan, Tao Han, Xu Zhang, Fenghui Li, Fei Tang, Hua Liu

**Affiliations:** ^1^ Department of Gastroenterology, The Third Central Hospital of Tianjin, Tianjin Key Laboratory of Extracorporeal Life Support for Critical Diseases, Artificial Cell Engineering Technology Research Center, Tianjin Institute of Hepatobiliary Disease, Tianjin, China; ^2^ Department of Gastroenterology, People’s Hospital Affiliated to Nankai University, Tianjin, China

**Keywords:** primary prophylaxis, rebleeding, endoscopic therapy, liver cirrhosis, esophagogastric variceal bleeding

## Abstract

**Aim:**

To identify the association between endoscopic primary prophylaxis and the risk of rebleeding in patients with liver cirrhosis receiving endoscopic therapy.

**Methods:**

This cohort study involved in 944 liver cirrhosis patients with esophagogastric variceal bleeding (EGVB) receiving endoscopic therapy. All participants were divided into two groups: rebleeding group (*n* = 425) and non-rebleeding group (*n* = 519) according to the occurrence of rebleeding in patients. Rebleeding indicated any bleeding after endoscopic therapy for the first bleeding of esophagogastric varices in liver cirrhosis patients. Univariate and multivariate logistic analyses were employed to identify the association between endoscopic primary prophylaxis and rebleeding in patients with liver cirrhosis after endoscopic therapy.

**Results:**

In total, 425 patients rebleeded at the end of the follow-up. The risk of rebleeding in patients with endoscopic primary prophylaxis decreased by 0.773 times (OR = 0.227, 95%CI: 0.139–0.372, *P *< 0.001) after adjusting covariables. Subgroups were divided according to the Child-Pugh (CP) score, and the results revealed that the risk of rebleeding in patients with endoscopic primary prophylaxis decreased by 0.858 times in Grade A patients (OR = 0.142, 95%CI: 0.066–0.304, *P *< 0.001) and 0.804 times in Grade B patients (OR = 0.196, 95%CI: 0.085–0.451, *P *< 0.001) compared with patients without endoscopic primary prophylaxis, but showed no difference in Grade C patients.

**Conclusion:**

Endoscopic primary prophylaxis was associated with a decreased risk of rebleeding in liver cirrhosis patients with EGVB after endoscopic therapy, which suggested that clinicians should pay more attention to endoscopic primary prophylaxis to prevent the occurrence of rebleeding in these patients.

## Introduction

Liver cirrhosis is a major cause of death all over the world, and its mortality rate is still on the rise (1). Portal hypertension is a serious complication of liver cirrhosis, which may result in esophagogastric variceal bleeding (EGVB) in patients (2). Esophagogastric varices were found in about 50% of newly diagnosed liver cirrhosis patients (3). Every year, 12% of liver cirrhosis patients suffered from the occurrence of first variceal bleeding (4). Previous studies have also revealed that the mortality of acute variceal bleeding in patients with liver cirrhosis was about 15%–20% within 6 weeks and 40% within 1 year (5, 6). Worse still, 60% of liver cirrhosis patients surviving EGVB may have a higher risk of esophagogastric variceal rebleeding (EGVR) and the mortality rate is up to 33% (7). There is an urgent need to improve the management of liver cirrhosis patients with EGVB.

Currently, the first-line standard treatment for liver cirrhosis with variceal bleeding is the combination of vasoactive drug therapy and endoscopic methods of hemostasis (8, 9). Several treatment methods were applied for varices and variceal hemorrhage, including transjugular intrahepatic portosystemic shunt (TIPS), and liver transplantation (10). The lack of liver donors and high medical costs limit the application of liver transplantation and TIPS may cause the reduction of liver function in patients (11). Additionally, the risk of rebleeding and mortality due to EGVB remains high despite the improvements in these therapies (12). Recently, primary prophylaxis was recommended for liver cirrhosis patients with esophagogastric varices, which has been reported to prevent the first bleeding in liver cirrhosis patients (13, 14). Non-selective beta-blockers (NSBBs) are widely utilized for primary and secondary prophylaxis of esophageal variceal bleeding in liver cirrhosis, which can reduce the portal vein velocity (15). Antibiotic prophylaxis was applied in cirrhosis patients, especially Child-Pugh B and C (16). Endoscopic therapy to ligate the varices was also a choice for the prevention and treatment of bleeding in cirrhosis patients, but the association of endoscopic therapy as primary prophylaxis and rebleeding in patients with liver cirrhosis and EGVB was still unclear.

The purpose of this study was to identify whether endoscopic primary prophylaxis had any association with the risk of rebleeding in patients with liver cirrhosis, which might provide a reference for preventing the occurrence of rebleeding in patients with liver cirrhosis complicated with EGVB using endoscopic therapy.

## Methods

### Study Design and Population

In this cohort study, the data of 944 liver cirrhosis patients with EGVB receiving endoscopic therapy after first bleeding were collected in the Third Central Hospital of Tianjin from Jan 2015 to June 2020. EGVB was caused by portal hypertension due to liver cirrhosis. The diagnosis of liver cirrhosis was based on the pathological examination or clinical diagnosis according to physical signs, ultrasound, computed tomography (CT), or biochemical indices (17). General gastroscopy was applied for the determination of esophageal varices. Esophageal varices were divided into three: mild (G1): esophageal varices were linear or slightly circuitous, without any red sign. Moderate (G2): esophageal varices were linear or slightly tortuous, with a red sign or serpentine protuberance but no red sign. Severe (G3): esophageal varices were serpentine and tortuous, with a red sign, or they were in the form of beads, nodules, or tumors (with or without any red sign) (18). Gastric varices were classified as gastroesophageal varices (GOV) and isolated gastric varices (IGV) (19). Variceal bleeding was defined according to Baveno VI criteria (8). The informed consents were collected from the participants, and our study was approved by the Ethics Committee of the Third Central Hospital of Tianjin (IRB2021-028-01).

### Endoscopic Treatment

In our study, endoscopic treatment was applied in the primary prophylaxis and treatment after first bleeding in liver cirrhosis. Endoscopy was conducted by the use of OLYMPUS 260 or 290 (Japan). For patients with gastric varices, lauromacrogol and tissue adhesive injection were applied. For patients with esophageal varices, endoscopic variceal ligation (EVL) was performed with a multiband ligator (Wilson Cook, USA). For patients with gastroesophageal varices, lauromacrogol and tissue adhesive injection, combined with EVL, were used.

### Data Collection

The baseline data of patients were collected: They are as follows: gender, age (year), the pathogenesis of liver cirrhosis [Hepatitis B Virus (HBV), hepatitis C virus (HCV), alcoholic, cryptogenic, autoimmune, and other reasons], time from admission to receiving endoscopic treatment (<6 h, ≥6 h and <12 h, ≥12 h and <24 h, and ≥24 h and <48 h), patients receiving painless endoscopy (yes or no), the frequency of endoscopic treatment, whether having a CT portosystemic shunt, prothrombin activity (PTA, %), international normalized ratio (INR), white blood cell (WBC, 10⁹/l), neutrophil (NEUT, 10⁹ g/l), lymphocyte (10⁹ g/l), hemoglobin (Hb, g/l), platelet (PLT, 10⁹ g/l), albumin (ALB, g/l), alanine transaminase (ALT, U/l), aspartate aminotransferase (AST, U/l), total bilirubin (TBIL, μmol/l), creatinine (CR, μmol/l), sodium (Na), alpha fetoprotein (AFP, μg/l), varices invalid or alleviated after treatment, whether having portal vein thrombosis, the width of the portal vein (mm), spleen thickness (mm), ascites grade, hepatic encephalopathy, shock, infection, and antibiotics using status, Child-Pugh (CP) score (Grade A: 5–6 points, Grade B: 7–9 points and Grade C: ≥10 points), and whether there is any occurrence of rebleeding.

### Outcome Variable

Inpatient or outpatient follow-up was performed every 3 months after the treatment of the first bleeding, until the variceal obliteration. According to the Baveno VI document, a combination of either a 3 g/dl drop in hemoglobin, fresh hematemesis, or death within 5 days is defined as rebleeding.

### Statistical Analysis

Statistical analyses were performed using SAS 9.4 software. All statistical analyses were subjected to a two-side test. The Shapiro test was conducted to evaluate the normality of measurement data. The measurement data with normal distribution were displayed as mean ± standard deviation (SD), and comparisons between groups were based on the independent sample t-test. The measurement data with non-normal distribution were depicted as M (Q_1_, Q_3_), and the differences between groups were evaluated using the Mann–Whitney U test. The enumeration data were described as *n* (%), and Chi-square (*χ*^2^) test or Fisher's exact probability method were used for making comparisons between groups. Variables with statistical differences between the rebleeding group and the non-rebleeding group were adjusted as covariates in multivariate logistic regression analysis to identify the association of endoscopic primary prophylaxis and rebleeding in patients with liver cirrhosis. The univariate model was the crude model, the multivariable model^a^ adjusted for painless endoscopic therapy and endoscopic therapy frequency. In the multivariate model ^b^, painless endoscopic therapy, endoscopic therapy frequency, lymphocyte, Hb, AST, varices status, spleen thickness, ascites grade, and shock were adjusted. A score of *P *< 0.05 was considered statistically significant.

## Results

### The Baseline Characteristics of All Participants

**Figure 1:**
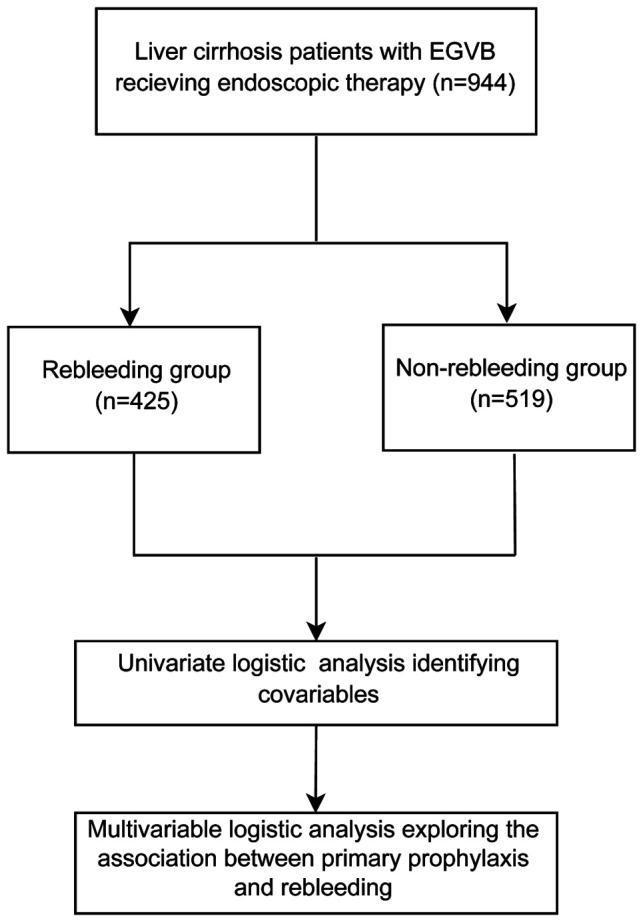
The analysis process of our study.

This study involved 944 patients with liver cirrhosis (Figure 1). All participants were divided into two groups, the rebleeding group (*n* = 425) and the non-rebleeding group (*n* = 519), according to the occurrence of rebleeding in patients receiving endoscopic therapy. The average age of all participants was 57.36 ± 11.08 years. Among them, 610 (64.62%) were males and 334 (35.38%) were females. A total of 198 (20.97%) patients had endoscopic primary prophylaxis. The pathogenesis of 435 (46.08%) patients was HBV, 76 (8.05%) were HCV, 162 (17.16%) were alcoholic, 77 (8.16%) were cryptogenic, 120 were autoimmune (12.71%), and 74 were others (7.84%). A total of 337 (35.70%) patients received painless endoscopy, and the median frequencies of endoscopic treatment in all patients were 2 (1, 4) times. According to the CP scores, 435 (46.08%) patients were in Grade A, 385 (40.78%) participants were in Grade B, and 124 (13.14%) patients were in Grade C. There were 425 (45.02%) patients in the rebleeding group and 519 (54.98%) patients in the non-rebleeding group (Table 1).

**Table 1:** The baseline characteristics of all participants.

Characteristic	Description (*n* = 944)
Gender, *n* (%)	
Male	610 (64.62)
Female	334 (35.38)
Age, Mean ± SD	57.36 ± 11.08
Primary prophylaxis, *n* (%)	
No	746 (79.03)
Yes	198 (20.97)
Pathogenesis, *n* (%)	
HBV	435 (46.08)
HCV	76 (8.05)
Alcoholic	162 (17.16)
Cryptogenic	77 (8.16)
Autoimmune	120 (12.71)
Others	74 (7.84)
Time from admission to hospital to endoscopic treatment, *n* (%)	
<6 h	140 (14.85)
≥6 h and <12 h	98 (10.39)
≥12 h and <24 h	260 (27.57)
≥24 h and <48 h	445 (47.19)
Unknown	1 (0.11)
Painless endoscopy, *n* (%)	
No	607 (64.30)
Yes	337 (35.70)
Frequency of endoscopic treatment, M (Q_1,_Q_3_)	2 (1,4)
CT portosystemic shunt, *n* (%)	
No	831 (88.03)
Yes	113 (11.97)
CP score, *n* (%)	
Grade A	435 (46.08)
Grade B	385 (40.78)
Grade C	124 (13.14)
Group, *n* (%)	
Rebleeding	519 (54.98)
Non-rebleeding	425 (45.02)

*HBV: Hepatitis B Virus, HCV: hepatitis C virus, CP: Child-Pugh.*

### Comparisons of the Data in Patients Between the Rebleeding Group and the Non-Rebleeding Group

As observed in Table 2, the proportions of patients receiving endoscopic primary prophylaxis (10.35% vs 29.67%, *χ*^2 ^= 52.616, *P *< 0.001) and painless endoscopy (25.41% vs 44.12%, *χ*^2 ^= 35.639, *P *< 0.001) in the rebleeding group were lower than those in the non-rebleeding group. The proportions of patients with varices becoming invalid after endoscopic therapy (41.65% vs 10.79%, *χ*^2 ^= 119.677, *P *< 0.001) and shock (3.53 vs 1.16, *χ*^2 ^= 6.051, *P *= 0.014) were significantly higher in the rebleeding group than those in the non-rebleeding group. The frequencies of endoscopic therapy (4 times vs 2 times, Z = 12.932, *P *< 0.001), the average lymphocyte level (14.60 10⁹ g/l vs 12.15 10⁹ g/l, Z = 2.580, *P *= 0.010), the mean spleen thickness (50.72 mm vs 48.96 mm, t = −2.000, *P *= 0.046), and ascites grade (Z = −2.106, *P *= 0.035) were higher in the rebleeding group than in the non-rebleeding group. The mean level of Hb (88.98 g/l vs 94.79 g/l, *P *< 0.001) and average level of AST (33.00 U/l vs 35.00 U/l, *P *= 0.025) were lower in the rebleeding group than in the non-rebleeding group (Table 2).

**Table 2:** Comparisons of the data in patients between two groups.

Characteristic	Total (*n* = 944)	Group		Statistical magnitude	*P*
		Non-rebleeding (*n* = 519)	Rebleeding (*n* = 425)		
Gender, *n* (%)				*χ*^2 ^= 0.105	0.746
Male	610 (64.62)	333 (64.16)	277 (65.18)		
Female	334 (35.38)	186 (35.84)	148 (34.82)		
Age, Mean ± SD	57.36 ± 11.08	57.63 ± 11.08	57.04 ± 11.08	t = 0.820	0.415
Primary prophylaxis, *n* (%)				χ^2 ^= 52.616	<0.001
No	746 (79.03)	365 (70.33)	381 (89.65)		
Yes	198 (20.97)	154 (29.67)	44 (10.35)		
Pathogenesis, *n* (%)				χ^2 ^= 8.434	0.134
HBV	435 (46.08)	254 (48.94)	181 (42.59)		
HCV	76 (8.05)	37 (7.13)	39 (9.18)		
Alcoholic	162 (17.16)	88 (16.96)	74 (17.41)		
Cryptogenic	77 (8.16)	43 (8.29)	34 (8.00)		
Autoimmune	120 (12.71)	54 (10.40)	66 (15.53)		
Others	74 (7.84)	43 (8.29)	31 (7.29)		
Time from admission to hospital to endoscopic treatment, *n* (%)				Z = −0.844	0.399
<6 h	140 (14.85)	82 (15.83)	58 (13.65)		
≥6 h and <12 h	98 (10.39)	38 (7.34)	60 (14.12)		
≥12 h and <24 h	260 (27.57)	148 (28.57)	112 (26.35)		
≥24 h and <48 h	445 (47.19)	250 (48.26)	195 (45.88)		
Unknown	1 (0.11)	1 (0.19)	0 (0)		
Painless endoscopy, *n* (%)				χ^2 ^= 35.639	<0.001
No	607 (64.30)	290 (55.88)	317 (74.59)		
Yes	337 (35.70)	229 (44.12)	108 (25.41)		
Frequency of endoscopic treatment, M (Q_1,_Q_3_)	2 (1,4)	2 (1,3)	4 (2,5)	Z = 12.932	<0.001
CT portosystemic shunt, *n* (%)				χ^2 ^= 0.691	0.406
No	831 (88.03)	461 (88.82)	370 (87.06)		
Yes	113 (11.97)	58 (11.18)	55 (12.94)		
PTA, Mean ± SD	67.63 ± 17.46	67.63 ± 17.74	67.63 ± 17.14	t = 0.001	0.999
INR, Mean ± SD	1.38 ± 0.36	1.38 ± 0.40	1.37 ± 0.31	t = 0.510	0.611
WBC, M (Q_1_,Q_3_)	4.12 (2.80,6.29)	3.97 (2.81,5.92)	4.28 (2.79,6.58)	Z = 1.359	0.174
NEUT, Mean ± SD	70.54 ± 10.97	70.60 ± 10.91	70.45 ± 11.06	t = 0.210	0.834
Lymphocyte, M (Q_1_,Q_3_)	13.00 (1.25,21.00)	12.15 (1.01,20.60)	14.60 (2.50,22.00)	Z = 2.580	0.010
HB, Mean ± SD	92.17 ± 26.33	94.79 ± 26.42	88.98 ± 25.90	t = 3.390	<0.001
PLT, M (Q_1_,Q_3_)	75.00 (54.00,103.50)	73.00(53.00,101.00)	77.00(56.00,110.00)	Z = 1.891	0.059
ALB, Mean ± SD	32.96 ± 6.30	32.86 ± 6.43	33.09 ± 6.15	t = −0.570	0.569
ALT, M (Q_1_,Q_3_)	26.00 (17.00,38.00)	26.00 (17.00,40.00)	26.00 (16.00,37.00)	Z = −1.152	0.249
AST, M (Q_1_,Q_3_)	34.00 (22.00,54.00)	35.00 (24.00,57.00)	33.00 (21.00,50.00)	Z = −2.236	0.025
TBIL, M (Q_1_,Q_3_)	21.00 (14.60,33.80)	21.10 (15.10,33.50)	20.80 (14.30,33.90)	Z = −0.722	0.470
CR, M (Q_1_,Q_3_)	65.00 (55.00,78.00)	66.00 (55.00,80.00)	64.00 (54.00,77.00)	Z = −0.546	0.585
Na, Mean ± SD	138.88 ± 5.35	138.93 ± 6.11	138.81 ± 4.25	t = 0.340	0.733
AFP, M (Q_1_,Q_3_)	3.24 (1.97,6.27)	3.39 (1.99,6.89)	3.05 (1.94,5.82)	Z = −1.538	0.124
Varices, *n* (%)				χ^2 ^= 119.677	<0.001
Invalid after treatment	233 (24.68)	56 (10.79)	177 (41.65)		
Alleviated after treatment	711 (75.32)	463 (89.21)	248 (58.35)		
Portal vein thrombosis, *n* (%)				χ^2 ^= 1.326	0.249
No	686 (72.67)	385 (74.18)	301 (70.82)		
Yes	258 (27.33)	134 (25.82)	124 (29.18)		
width of portal vein, Mean ± SD	14.03 ± 1.92	13.92 ± 1.77	14.16 ± 2.08	t = −1.940	0.053
spleen thickness, Mean ± SD	49.76 ± 13.50	48.96 ± 13.13	50.72 ± 13.88	t = −2.000	0.046
ascites grade, *n* (%)				Z = −2.106	0.035
No	399 (42.36)	206 (39.77)	193 (45.52)		
A few	382 (40.55)	213 (41.12)	169 (39.86)		
Moderate or too many	161 (17.09)	99 (19.11)	62 (14.62)		
Hepatic encephalopathy., *n* (%)				χ^2 ^= 0.092	0.762
No	919 (97.35)	506 (97.50)	413 (97.18)		
Yes	25 (2.65)	13 (2.50)	12 (2.82)		
Shock, *n* (%)				χ^2 ^= 6.051	0.014
No	923 (97.78)	513 (98.84)	410 (96.47)		
Yes	21 (2.22)	6 (1.16)	15 (3.53)		
Infection, *n* (%)				χ^2 ^= 0.435	0.510
No	542 (57.42)	293 (56.45)	249 (58.59)		
Yes	402 (42.58)	226 (43.55)	176 (41.41)		
Antibiotics, *n* (%)				χ^2 ^= 0.091	0.763
No	260 (27.54)	145 (27.94)	115 (27.06)		
Yes	684 (72.46)	374 (72.06)	310 (72.94)		

*HBV: Hepatitis B Virus, HCV: hepatitis C virus, PTA: prothrombin activity, INR: international normalized ratio, WBC: white blood cell, NEUT: neutrophil, Hb: hemoglobin, PLT: platelet, ALB: albumin, ALT: alanine transaminase, AST: aspartate aminotransferase, TBIL: total bilirubin, CR: creatinine, Na: sodium, AFP: alpha fetoprotein, CT: computed tomography.*

### Association Between Endoscopic Primary Prophylaxis and Rebleeding

According to the results in univariate and multivariate analyses, the data delineated that the risk of rebleeding after endoscopic therapy in patients with endoscopic primary prophylaxis was decreased by 0.733 times compared with patients without endoscopic primary prophylaxis (OR = 0.267, 95%CI: 0.185–0.385, *P *< 0.001). After adjusting for variables including painless endoscopic therapy and endoscopic therapy frequency, patients with endoscopic primary prophylaxis were associated with a 0.226-fold decrease of risk of rebleeding compared with patients without endoscopic primary prophylaxis (OR = 0.226, 95%CI:0.150–0.339, *P *< 0.001). After adjusting for variables including painless endoscopic therapy, endoscopic therapy frequency, lymphocyte, Hb, AST, varices status, spleen thickness, ascites grade and shock, the risk of rebleeding in patients with endoscopic primary prophylaxis was decreased by 0.773 times (OR = 0.227, 95%CI: 0.139–0.372, *P *< 0.001) (Table 3).

**Table 3:** Univariate and multivariate analyses of associations between primary prophylaxis and rebleeding.

Characteristic	Univariate		Multivariate^a^		Multivariate^b^	
	*P*	OR (95%CI)	*P*	OR (95%CI)	*P*	OR (95%CI)
Primary prophylaxis						
No		Ref		Ref		Ref
Yes	<0.001	0.267 (0.185-0.385)	<0.001	0.226 (0.150-0.339)	<0.001	0.227 (0.139-0.372)

^a^ *Adjusting variables, including those for painless endoscopic therapy and endoscopic therapy frequency.*

^b^ *Adjusting variables for painless endoscopic therapy, endoscopic therapy frequency, lymphocyte, Hb, AST, varices status, spleen thickness, ascites grade, and shock.*

### Subgroup Analysis of the Association Between Endoscopic Primary Prophylaxis and Rebleeding in Patients With a Different CP Score

All subjects were divided into Grade A, Grade B, and Grade C groups according to their CP score. As depicted in Table 4, in the Grade A group, the risk of rebleeding decreased by 0.806 times in patients with endoscopic primary prophylaxis compared with patients without endoscopic primary prophylaxis (OR = 0.194, 95%CI: 0.117–0.323, *P *< 0.001). After adjusting for variables including painless endoscopic therapy, endoscopic therapy frequency, lymphocyte, Hb, AST, varices status, spleen thickness, ascites grade and shock, the risk of rebleeding after endoscopic therapy in patients with endoscopic primary prophylaxis decreased by 0.858 times in comparison with those without endoscopic primary prophylaxis (OR = 0.142, 95%CI: 0.066–0.304, *P *< 0.001). As for patients in the Grade B group, endoscopic primary prophylaxis decreased the risk of rebleeding by 0.738 times compared with those without endoscopic primary prophylaxis (OR = 0.262, 95%CI: 0.140–0.490, *P *< 0.001). After adjusting for variables such as painless, endoscopic therapy frequency, lymphocyte, Hb, AST, varicose veins, splenic thickness, ascites grade, and shock, endoscopic primary prophylaxis decreased the risk of rebleeding after endoscopic therapy by 0.804 times compared with those without endoscopic primary prophylaxis (OR = 0.196, 95%CI: 0.085–0.451, *P *< 0.001). In the Grade C group, endoscopic primary prophylaxis showed no statistical difference on rebleeding risk in patients (*P *> 0.05).

**Table 4:** Subgroup analysis of the association between primary prophylaxis and rebleeding.

CP score	Univariate		Multivariate	
	OR(95%CI)	*P*	OR(95%CI)^a^	*P*
Grade A	0.194 (0.117-0.323)	<0.001	0.142 (0.066-0.304)	<0.001
Grade B	0.262 (0.140-0.490)	<0.001	0.196 (0.085-0.451)	<0.001
Grade C	1.244 (0.421-3.678)	0.693	1.719 (0.453-6.533)	0.426

*CP: Child-Pugh.*

^a^ *Adjusting variables for painless endoscopic therapy, endoscopic therapy frequency, lymphocyte, Hb, AST, varices status, spleen thickness, ascites grade, and shock.*

## Discussion

In this study, the data of 944 liver cirrhosis patients with EGVB receiving endoscopic therapy were collected to examine whether endoscopic primary prophylaxis had an impact on the rebleeding risk in liver cirrhosis patients with EGVB after endoscopic therapy. The data revealed that endoscopic primary prophylaxis decreased the risk of rebleeding in liver cirrhosis patients with EGVB after endoscopic therapy. Additionally, for patients with a CP score in Grade A and Grade B, the risk of rebleeding reduced by endoscopic primary prophylaxis, but in Grade C, there was no difference in the risk of rebleeding in patients irrespective of whether they received endoscopic primary prophylaxis. The findings of our study might provide a reference for the use of endoscopic primary prophylaxis in the clinic.

Variceal bleeding is a serious complication of liver cirrhosis with a high risk of mortality (20). Due to the compression of regenerated nodular tissue on venous vessels in liver fibrosis patients, the vessels were distorted, and the pressure on hepatic sinus and the terminal portal vein increased, thereby inducing EGVB (21). In our study, the pathogeneses of patients with liver cirrhosis were mainly HBV, HCV, alcoholic, cryptogenic, and autoimmune hepatitis. Liver cirrhosis patients suffered from liver cell degeneration and necrosis, the collapse of the hepatic lobular fibrous scaffold, and irregular regeneration of liver cells. At the same time, inflammation and other pathogenic factors activated hepatic astrocytes and promoted the transformation of hepatic cells and bile duct cells into interstitial cells. Collagen synthesis in the liver increased and degradation reduced. Then, a large amount of collagen was deposited in the Disse space, which narrowed the hepatic sinuses, leading to a reduction of hepatic sinusoids and a compression of hepatic sinusoids and hepatic venous systems. Therefore, the portal vein blood flow into the hepatic sinuses and the outflow tract of the hepatic vein after the sinus were blocked, which increased the vascular resistance and elevated the risk of portal hypertension. The gastric coronary vein of the portal vein system was anastomosed with the azygos vein of the esophageal vein of the vena cava system at the lower esophagus and stomach fundus, forming esophagogastric varices.

Previous researchers have made efforts to make an in-depth study of the pathogenesis of liver cirrhosis with portal hypertension, and new technologies have led to notable advances in treating EGVB. However, the mortality rate due to EGVB is still unfortunately high despite the application of current standards of treatment (22). Given the poor prognosis of EGVB patients, the primary prophylaxis of it in patients with esophagogastric varices has been widely proposed in recent years. EVL is recommended as a prevention method of primary prophylaxis for the first bleeding episode from esophagogastric varices (23). Endoscopic treatment of high risk lesions reduces the risk of bleeding and the need for surgery (24). EVL is an effective and safe treatment for the prevention of upper gastrointestinal bleeding in patients with portal hypertension (25). Another multicenter randomized-controlled trial revealed that as one of the primary prophylaxis, EVL is an effective method for preventing variceal hemorrhage in liver cirrhosis patients (26). In this study, we found that endoscopic primary prophylaxis using EVL was associated with a decreased risk of rebleeding in liver cirrhosis patients with EGVB after endoscopic therapy.

The CP classification was applied to assess the hepatic function according to the risk stratifications of ascites, encephalopathy, serum ALB, TBIL, and prothrombin time (27). The classification was graded based on the total score: Grade A: 5–6 points, Grade B: 7–9 points, Grade C: 10 points, or above. A previous study found that the CP score was an independent risk factor for immediate bleeding after colonoscopic polypectomy in liver cirrhosis patients, and the bleeding risk was higher in Grade C patients than in Grade A patients (28). Another large-scale cohort study also depicted that the 1-year and 3-year survival rates were 90.2% and 75.3% for Grade A, 73.5% and 53.9% for Grade B, and 41.9% and 28.9% for Grade C, indicating that patients in Grade C were associated with a poorer prognosis (29). In this study, subgroup analysis was performed to screen out patients who were found suitable for primary prophylaxis. The results demonstrated that endoscopic primary prophylaxis could decrease the risk of rebleeding in Grade A and Grade B patients, suggesting that endoscopic primary prophylaxis might be suitable for patients in these groups. This may be due to the fact that patients in Grade A and Grade B had better liver function and a longer survival time with appropriate treatment (30). Bleeding is a serious threat to the lives of patients, and primary prophylaxis decreased the risk of bleeding and caused little side effects in the recovery process in those patients (23). Additionally, the effect of endoscopic primary prophylaxis in decreasing the risk of rebleeding was better in the Grade A group than in the Grade B group. Endoscopic primary prophylaxis had no significant effects for Grade C patients because these patients had poor coagulation function, liver function, and nutritional status; the endoscopic treatment may increase the risk of bleeding, and it is difficult for them to recover after surgery (31). Therefore, clinicians might apply endoscopic primary prophylaxis based on the Child Pugh of patients.

The present study identified that endoscopic primary prophylaxis was associated with a reduced risk of rebleeding after endoscopic therapy in patients with cirrhosis complicated with EGVB. Endoscopic primary prophylaxis was performed before the first bleeding in liver cirrhosis patients with esophagogastric varices. Patients receiving endoscopic primary prophylaxis *via* endoscopic treatment had reduced varices, and the rebleeding risk decreased. Also, the volume of rebleeding reduced and the therapeutic effect was better (13). The risk of rebleeding after endoscopy is mainly related to the level of portal hypertension, the severity of varices, and the status of coagulation function in patients (32). Based on these results, we recommend endoscopic primary prophylaxis for patients with cirrhosis complicated with esophagogastric varices, rather than waiting for emergency treatment after the first episode of variceal bleeding.

This study was a case-control study including a large sample size to evaluate the association between endoscopic primary prophylaxis and rebleeding after endoscopic therapy in liver cirrhosis patients with EGVB. The variables were collected after admission to hospital, which decreased the recall bias. The first limitation of this study was that this was a single-center study. Was that the data of all participants from the time of endoscopic treatment for first bleeding to rebleeding, which might have association with the risk of rebleeding. In the future, the findings of our study should be validated by multicenter studies with a large sample size.

## Conclusions

Our study collected the data of 944 liver cirrhosis patients with EGVB receiving endoscopic therapy to find whether endoscopic primary prophylaxis was associated with the risk of rebleeding after endoscopic therapy in these patients. The results showed that endoscopic primary prophylaxis was associated with a decreased risk of rebleeding in liver cirrhosis patients with EGVB after endoscopic therapy. These findings will serve to remind clinicians to pay more attention to endoscopic primary prophylaxis in liver cirrhosis patients with esophagogastric varices and provide proper and timely treatment in those patients. For patients in Grade C with poor liver function, endoscopic primary prophylaxis should be done with caution.

## Data Availability

The raw data supporting the conclusions of this article will be made available by the authors, without undue reservation.
